# Cognitive Profiles and Hub Vulnerability in Parkinson's Disease

**DOI:** 10.3389/fneur.2018.00482

**Published:** 2018-06-20

**Authors:** Sue-Jin Lin, Tobias R. Baumeister, Saurabh Garg, Martin J. McKeown

**Affiliations:** ^1^Graduate Program in Neuroscience, University of British Columbia, Vancouver, BC, Canada; ^2^Pacific Parkinson's Research Centre, University of British Columbia, Vancouver, BC, Canada; ^3^School of Biomedical Engineering, University of British Columbia, Vancouver, BC, Canada; ^4^Neurology, Faculty of Medicine, University of British Columbia, Vancouver, BC, Canada

**Keywords:** cognition, hubs, resting-state functional connectivity, canonical correlation analysis, graph theory, Parkinson's disease, functional segregation

## Abstract

The clinicopathological correlations between aspects of cognition, disease severity and imaging in Parkinson's Disease (PD) have been unclear. We studied cognitive profiles, demographics, and functional connectivity patterns derived from resting-state fMRI data (rsFC) in 31 PD subjects from the Parkinson's Progression Markers Initiative (PPMI) database. We also examined rsFC from 19 healthy subjects (HS) from the Pacific Parkinson's Research Centre. Graph theoretical measures were used to summarize the rsFC patterns. Canonical correlation analysis (CCA) was used to relate separate cognitive profiles in PD that were associated with disease severity and demographic measures as well as rsFC network measures. The CCA model relating cognition to demographics suggested female gender and education supported cognitive function in PD, age and depression scores were anti-correlated with overall cognition, and UPDRS had little influence on cognition. Alone, rsFC global network measures did not significantly differ between PD and controls, yet some nodal network measures, such as network segregation, were distinguishable between PD and HS in cortical “hub” regions. The CCA model relating cognition to rsFC global network values, which was not related to the other CCA model relating cognition to demographic information, suggested modularity, rich club coefficient, and transitivity was also broadly related to cognition in PD. Our results suggest that education, aging, comorbidity, and gender impact cognition more than overall disease severity in PD. Cortical “hub” regions are vulnerable in PD, and impairments of processing speed, attention, scanning abilities, and executive skills are related to enhanced functional segregation seen in PD.

## Introduction

Parkinson's disease (PD) is a neurodegenerative movement disorder resulting in motor symptoms of tremor, rigidity, bradykinesia, and postural instability. In addition to motor symptoms, non-motor deficits, especially cognitive impairments, have a major impact on quality of life in patients with PD. Patients with PD show cognitive deficits in several common domains such as attention, memory, visuospatial, and executive functions (1, 2). Older age, non-tremor subtype, and higher Unified Parkinson's Disease Rating Scale (UPDRS) scores are risk factors for the rapid overall cognitive decline (3), and specifically, information retrieval and visuospatial abilities can predict global cognitive impairments in PD (3). Rather than simply categorizing patients into cognition-intact and cognition-impaired subtypes, there is substantial heterogeneity of cognitive performance in PD, and “frontal,” “posterior,” and “mixed” subtypes have been proposed (4). Traditional research typically attempts to probe one aspect of cognition (e.g., attention) with one measure, but it is very difficult to test components of cognition in an isolated fashion. Hence performance on different cognitive tests often correlate with each other, and it is likely that novel analyses such as machine-learning approaches will be more suitable in establishing cognitive patterns in subjects with neurological disorders.

Many neuroimaging studies attempt to link cognitive deficits in PD and functional connectivity (FC) at rest. Impaired corticostriatal connectivity, resulting in decreased integration among the striatum, mesolimbic cortex, and sensorimotor cortex has been associated with some non-motor symptoms in PD such as mental “rigidity” (5). Overall global cognitive performance in PD is shown to be associated with decreased FC in widespread regions including the paracentral lobe, superior parietal lobe, occipital regions, inferior frontal gyrus, and superior temporal gyrus (6). Weakened FC in the frontoparietal network has been shown to be related to worsening executive function in PD with mild cognitive impairments (MCI) (7). Therefore, it is speculated that not only the frontostriatal connections, but whole-brain altered connectivity contributes to cognitive deficits in PD.

One of the strategies to characterize the whole brain FC is to apply *graph theory analysis* to summarize the overall network of connections between Regions of Interest (ROIs). These network characteristics summarize the whole brain FC globally and locally in terms of functional integration, segregation, and core/hub structures (8, 9). Several network measures have been linked to human intelligence and cognitive functions in healthy subjects (10, 11). In PD, regions such as the orbitofrontal regions and occipital pole show decreased node degree (i.e., fewer numbers of connections to/from a given region); while nodes with increased degree are located in the superior parietal, posterior cingulate cortex, supramarginal, and supplementary motor areas (12). Baggio et al. (13) discovered reduced FC in long-range connections (i.e., connections between frontal, occipital, and parietal areas) in both PD and PD with MCI; while graph theoretical measures representing network segregation in frontal areas were negatively correlated with attention/executive scores in PD with MCI, possibly as compensation/adaptation for impairments in long-range connections.

Previous work has suggested that “hub regions” may be particularly vulnerable to neurodegenerative processes (14). In healthy subjects, the insula and several frontal regions such as the superior frontal gyrus act as hubs and may facilitate cognitive processes to an even greater extent than other structures traditionally thought to be critical for many cognitive processes, such as the caudate (15, 16). In PD, there is a tendency of failure in hub regions and non-hub regions' connectivity becomes more prominent than that in healthy subjects (17). Reduced importance of hubs' connectivity and increased importance of non-hub regions' connectivity in prefrontal areas has been observed in PD with MCI (13). Even without MCI, in PD the insula may lose connectivity, but there may be an enhanced role of the caudate (17).

In this study, we aimed to investigate (1) the cognitive profile associated with demographics in PD, (2) the functional connectivity differences between PD and healthy subjects measured by graph theory analysis (which may or may not be related to cognitive deficits), and (3) the cognitive profiles associated with altered graphical measures from rsFC in PD. We have previously demonstrated the value of comparing cognitive scores and demographics using canonical correlation analysis (CCA) in patient populations (18); In addition, instead of only investigating FC in the frontostriatal loops, we studied whole-brain connectivity. Finally, we utilized both univariate and multivariate analyses to explore the associations between FC and cognitive performance in PD.

## Materials and methods

### Subjects

Thirty-one subjects with PD who enrolled in the Parkinson's Progression Marker Initiative (PPMI) were included in the study. All subjects underwent neuropsychological assessments and imaging scans with T1-weighted MRI and resting-state fMRI (rsfMRI). All data in this study were acquired at baseline. The inclusion criteria required subjects must show at least two of the following: resting tremor, bradykinesia, rigidity or either asymmetric resting tremor or asymmetric bradykinesia. Subjects had a diagnosis of PD for 2 years or less, Hoehn and Yahr stage I or II, off medication, age 30 years or older at diagnosis, and ability to provide written consent. Exclusion criteria included atypical PD syndromes, taking any PD medication, taking levodopa or dopamine agonists prior to baseline for more than a total of 60 days, dementia [screened by Montreal Cognitive Assessment (MoCA)], and any other medical or psychiatric condition or lab abnormalities. Demographics were shown in Table 1.

**Table 1:** Demographics, clinical data, and cognitive scores in Parkinson's and healthy subjects.

	**Parkinson's subject (mean ± std)**	**Healthy subject (mean ± std)**
**DEMOGRAPHICS AND CLINICAL DATA**		
Gender	10females±21males	9 females, 10 males
Age	60.95 ± 9.7	56.12 ± 16.9
UPDRS	15.51 ± 11.3	No data
Depression	5.19 ± 1.0	No data
Education in years	16.81 ± 2.9	No data
**COGNITIVE SCORES**		
MOCA	27.52 ± 2.1	27.39 ± 1.6*
BJLOTOT	25.23 ± 3.9	No data
HVLTTOT	23.32 ± 5.5	No data
HVLTDELAY	8.32 ± 2.8	No data
DVT-HVLTTOTAL	44.32 ± 14.4	No data
DVT-HVLTDELAY	49.23 ± 16.1	No data
DVT-HVLTRETENTION	51.87 ± 14.4	No data
LNS-RAW	10.26 ± 2.7	No data
SFCOM	47.35 ± 10.8	No data
SFVEG	13.35 ± 4.0	No data
SFANI	21.19 ± 5.0	No data
SFFRU	12.81 ± 3.8	No data
SDMT	41.10 ± 9.3	No data

UPDRS, Unified Parkinson's Disease Rating Scale; MOCA, Montreal Cognitive Assessment; BJLOTOT, Bento Line Orientation Total Score; HVLTTOT, Hopkins Verbal Learning Test-Revised Total Score; HVLTDELAY, HVLT Delayed Recall Score; DVT-HVLTTOTAL, standardized HVLT Total Score; DVT-HVLTDELAY, standardized HVLT Delayed Recall Score; DVT-HVLTRETENTION, standardized HVLT Recognition Trial Score; LNS-RAW, raw Letter-Number Sequencing Test Score; SFCOM, Sematic Fluency Test—combination; SFVEG, Sematic Fluency Test—vegetable trial; SFANI, Sematic Fluency Test—animal trial; SFFRU, Sematic Fluency Test—fruit trial; SDMT, Symbol Digit Modalities Test. ^*^There is 1 missing data set.

For the analysis of cognitive profiles, all 31 subjects were included and three of them showed MCI. For imaging analysis, 23 subjects were included as 8 subjects had excessive imaging and motion artifacts and only one of the included subjects in connectivity analysis showed MCI.

For comparison, 19 age-matched healthy subjects (HS) were recruited in this study through Pacific Parkinson's Research Centre at UBC Hospital. None of the HS showed cognitive impairment screened by MoCA. Ethics approval was issued by the University of British Columbia's Research Ethics Board and all subjects provided written, informed consent.

### Neuropsychological assessments

The neuropsychological assessments performed on PD subjects have been previously described (19). In total, there were 13 scores from the assessments included MoCA, Benton Judgment of Line Orientation (BJLO), raw Hopkins Verbal Learning Test-Revised (HVLT), raw HVLT delayed recall, standardized HVLT, standardized HVLT delayed, standardized HVLT recognition trial, raw Letter-Number Sequencing test (LNS), Sematic Fluency Test—combination (SFCOM), Sematic Fluency Test—vegetable trial (SFVEG), Sematic Fluency Test—fruit trial (SFFRU), Sematic Fluency Test—animal trial (SFANI), and Symbol Digit Modalities Test (SDMT) scores (Table 1). Memory, visuospatial, and attention/executive functioning domains were measured, which were the most common affected domains in PD.

HS did not undergo neuropsychological assessments other than MoCA.

### Image acquisition

In PD subjects, a standardized MRI protocol on a 3 Tesla Siemens Trio Tim MR system was used. 3D T1-weighted structural images were acquired using MPRAGE GRAPPA sequence with Repetition Time (TR) 2,300 ms, Echo Time (TE) 2.98 ms, Field of View (FoV) 256 mm, and resolution 1 × 1 × 1 mm^3^. RsfMRI images were acquired using echo-planar imaging (EPI) to detect Blood Oxygenation Level Dependent (BOLD) contrast with 212 volumes, 40 slices in ascending direction, TR 2,400 ms, TE 25 ms, FoV 222 mm, and resolution 3.3 × 3.3 × 3.3 mm^3^.

All HS subjects underwent imaging scans in MRI research center at UBC Hospital with a Philips Achieva 3.0 Tesla MRI scanner. 3D T1-weighted images were acquired with TR 8 ms, TE 4 ms, FoV 256 mm, and resolution 1 × 1 × 1 mm^3^. RsfMRI data were acquired with EPI sequence and the following parameters: 186 volumes/dynamics, 36 slices in interleaved direction, TR 2,500 ms, TE 30 ms, FoV 240 mm, and resolution 3 × 3 × 3.97 mm^3^.

During rsfMRI scans, all subjects were instructed to rest quietly with their eyes closed and not to fall asleep.

### Data analysis

#### Cognitive scores and demographics

Canonical correlation analysis (CCA) was applied to establish the correlation between two sets of variables. CCA can be seen as an extension of linear regression and a simple machine learning method, which seeks the linear combinations of variables that maximize the correlation between two datasets. Details of the approach can be found in a previous study (18). Here, the two sets were the cognitive scores measured by neuropsychological assessments and the demographics of 31 PD subjects. The demographics set included gender (encoded as female 2 and male 1), age, UPDRS scores, depression, and years of education. The cognitive set included MoCA, BJLO, raw HVLT, raw HVLT delayed recall, standardized HVLT, standardized HVLT delayed, standardized HVLT recognition trial, raw LNS, SFCOM, and SDMT scores. As in sematic fluency, the combination trial should represent the performance of sematic fluency in animal, vegetable, and fruit trials, so we only included SFCOM instead of taking all 4 scores. All scores were normalized into *z*-scores before CCA. We defined the linear combination of cognitive variables as the cognitive profile associated with demographics (CP_dem_). The CCA was performed in a leave-one-out fashion to ensure robustness of our results, and reported the loadings (i.e., the correlation between transformed CCA data and raw scores) for each variable. If the 95% confidence intervals of each variable based on the cross-validation did not cross zero, we regarded this variable as a significant contributor to the CCA model. A permutation test was carried out with 1,000 iterations to evaluate the significance of the correlation in CCA.

#### Imaging preprocessing

Image processing steps were performed in subject's native space. The functions of slice-timing and motion correction from SPM8 (UCL, London) were used for fMRI preprocess and FLIRT toolbox from FSL 6.0 (FMRIB, Oxford) was used for registration. Moreover, fMRI images were rescaled to isotropic voxel size using a self-programmed script in Matlab (The MathWorks, Inc.), so all fMRI data of HS and PD were 3 × 3 × 3 and 3.3 × 3.3 × 3.3 mm^3^ resolution, respectively. Cortical parcellation of the high-resolution T1 image was done in Freesurfer version 5.3.0 (MGH, Boston) and 68 cortical and subcortical ROIs were included for connectivity analysis. We considered as many cortical regions as possible but excluded the ROIs that may have a poor signal-to-noise ratio (SNR) in fMRI due to partial volume effect such as the frontal pole and temporal pole. A brain mask was applied before registration to remove non-brain tissue and we registered the structural image to the mean fMRI image. The same transformation was subsequently applied to the ROI mask in order to obtain the ROIs in fMRI resolution. The ROIs acted as masks to determine the appropriate voxels making up the average ROI time courses. Finally, the fMRI time courses of these 68 ROIs were extracted using in-house scripts in Matlab and the data were detrended before connectivity analysis. Subjects who had translational and rotational head movements during data acquisition of more than 2 mm and 2 degrees respectively were excluded. In the end, 23 subjects were included in connectivity analysis.

#### Functional connectivity and graph theory analyses

Partial correlation analysis was conducted to generate a connectivity matrix for each subject in 23 PD and 19 HS, which resulted in a 68-by-68 matrix per subject. The Brain Connectivity Toolbox (BCT) (9) was used to compute graph theoretical measures. The partial correlation matrix was proportionally thresholded and binarized with the density of 15% to ensure equal density across subjects (20).

For global measures, global efficiency, transitivity, modularity, assortativity, characteristic path length, rich club coefficient (at level 6, which is the highest degree possible due to matrix sparsity) were computed (9, 21). These global measures summarized network characteristics of the entire brain network.

For local measures, betweenness centrality and local efficiency were computed (9, 21), which reflect nodal characteristics in the network. Definitions of these measures can be found in Supplementary Table 1.

Two sample *t*-tests were carried out to test whether graph theoretical measures were significantly different between PD and HS. Logistic LASSO (22) was further applied to test whether the graphical measures could be used to distinguish PD from HS, as well as indicating those measures with the largest contribution.

#### Brain-behavior analysis

##### Univariate approach: correlation analyses

In order to investigate the relations between graph theoretical measures and cognition, we utilized Spearman's correlation analysis exploring whether individual cognitive tests and/or CP_dem_ were correlated with graph theoretical measures in PD. For global measures, the correlation analyses were carried out on all the global measures against raw cognitive scores from individual tests as well as CP_dem_. For local measures, only ROIs that showed significant differences in *t*-tests were included in the analyses. The local measures of these ROIs were included to correlate with both raw cognitive scores and CP_dem_. Bonferroni correction was applied to correct for multiple comparisons.

##### Multivariate approach: canonical correlation between cognitive and graphical measures

We further tested whether graphical measures were correlated with cognitive performance in a multivariate manner using CCA. All global measures were concatenated to form one set and the other set included cognitive scores. Likewise, the local measures which showed differences in the *t*-tests were included as one set and cognitive scores were included as the other set. All variables were normalized into z-scores and then processed with CCA. This analysis returned a cognitive profile associated with graphical measures (CP_gt_). As before, the canonical loadings with leave-one-out cross validation of all variables were reported and a permutation test was carried out with 1,000 iterations to evaluate the significance of the correlation in CCA.

**Figure 1:**
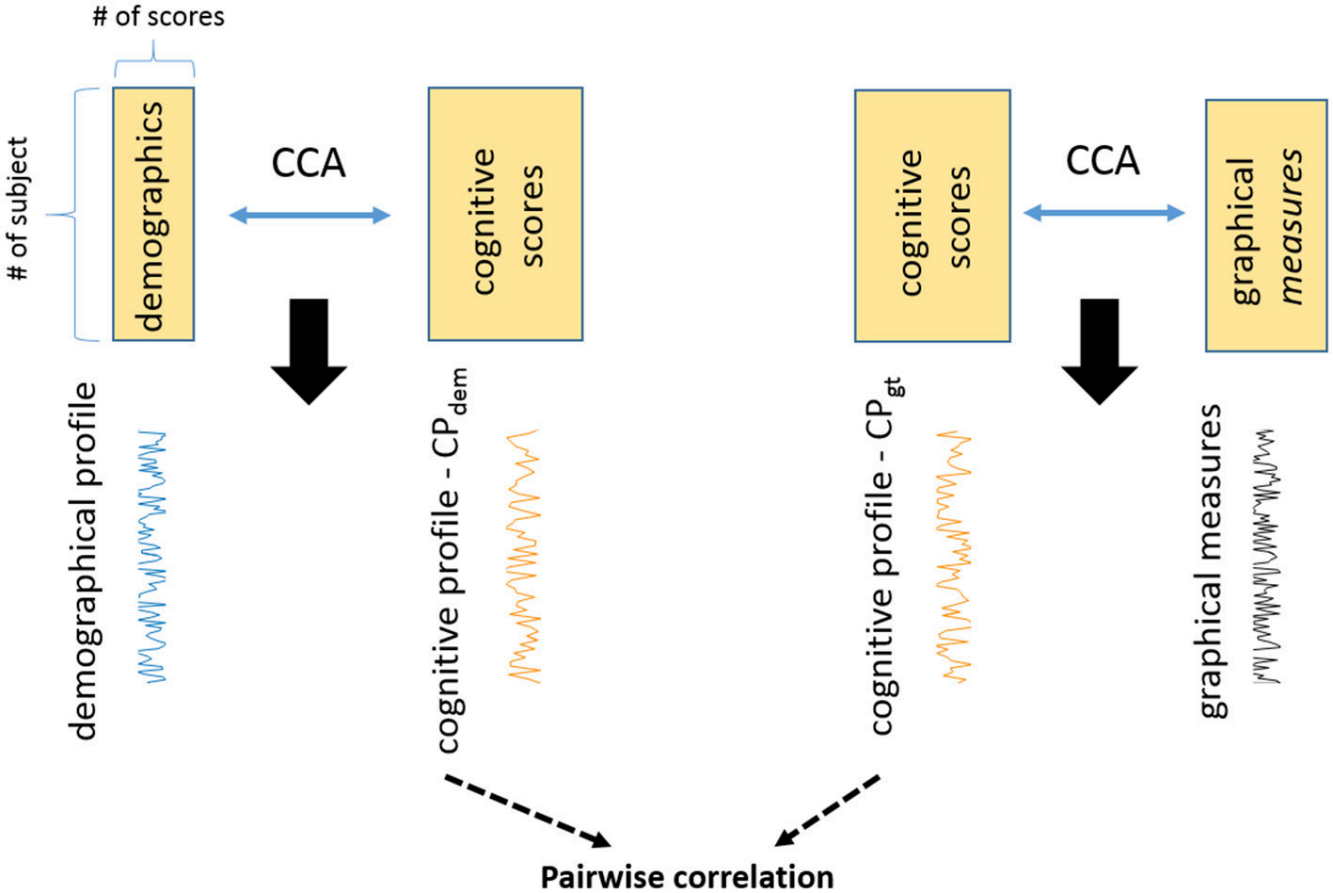
The study reveals two cognitive profiles in PD with CCA. One is the cognitive profile associated with demographics (CP_dem_). The other one is the cognitive profile associated with graphical measures (CP_gt_). These are two unique cognitive profiles and they are not related to each other (correlation = −0.06, *p* = 0.77).

Finally we assessed whether cognitive profiles associated with demographics and imaging features provided shared or unique information. Figure 1 shows a schematic diagram of two cognitive profiles.

## Results

### Cognitive profile associated with demographics

**Figure 2:**
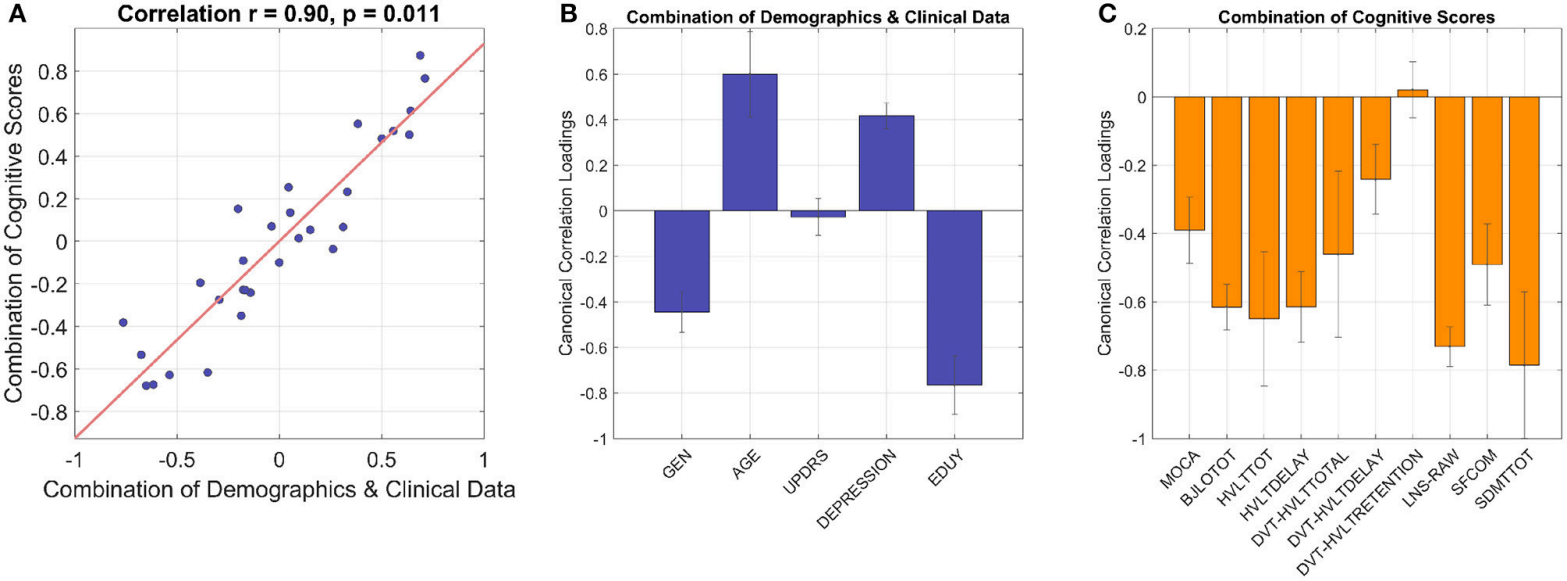
The CCA model shows inter-correlated cognitive patterns with demographics and clinical data. The linear combination of demographics/clinical data is significantly correlated with the linear combination of cognitive performances with correlation coefficient 0.90 and *p* 0.01 **(A)**, cross-validation with leave-one-out). **(B)** Gender, age, depression, and education have significant loadings in the combination of demographics and clinical data (i.e., 95% confidence interval does not cross zero as indicated by error bars). In the combination of cognitive scores **(C)**, all the variables show significant loadings except standardized HVLT Recognition Trial Score as the 95% confidence interval crosses zero. GEN, gender; EDUY, education in years; MOCA, Montreal Cognitive Assessment; BJLOTOT, Bento Line Orientation Total Score; HVLTTOT, Hopkins Verbal Learning Test-Revised Total Score; HVLTDELAY, HVLT Delayed Recall Score; DVT-HVLTTOTAL, standardized HVLT Total Score; DVT-HVLTDELAY, standardized HVLT Delayed Recall Score; DVT-HVLTRETENTION, standardized HVLT Recognition Trial Score; LNS-RAW, raw Letter-Number Sequencing Test Score; SFCOM, Sematic Fluency Test—combination; SDMTTOT, Symbol Digit Modalities Test total scores.

CCA revealed that demographics/clinical data were inter-correlated with cognitive scores (Figure 2). In the combination of demographics and clinical data, gender, age, depression, and education all showed significant canonical loadings, but UPDRS scores did not have an impact on this model. In the combination of cognitive scores, all variables demonstrated significant loadings except the standardized HVLT Recognition Trial Score. Variables with loadings the same sign were correlated with each other (i.e., negative loadings in demographics were positively correlated with negative loadings in cognitive scores) and anti-correlated with the variables that demonstrated the opposite sign. The two linear combinations of these two sets of variables were significantly correlated with each other with a correlation coefficient of 0.90, *p* = 0.011, and *p* = 0.015 in the permutation test.

The model demonstrated gender differences of cognitive performances in PD, where female subjects with more years of education were related to higher scores in almost all cognitive tests as they showed higher performance *z*-scores than males (Supplementary Figure 1). Further analyses supported this observation, as the mean *z*-scores of all cognitive tests/variables with significant CCA loadings were higher in female subjects than male subjects except the BJLO test (Supplementary Figure 1). Therefore, the variable *gender* in the model (shown in Figure 2) demonstrated an *overall common effect* to cognition across tests rather than specific patterns in individual tests. Similarly, education supported overall cognitive functions in PD. Meanwhile, higher age and higher scores in depression scale were anti-correlated with all cognitive scores which showed significant loadings, implying that aging and PD comorbidities such as depression worsened cognitive abilities.

### Graphical measures

**Figure 3:**
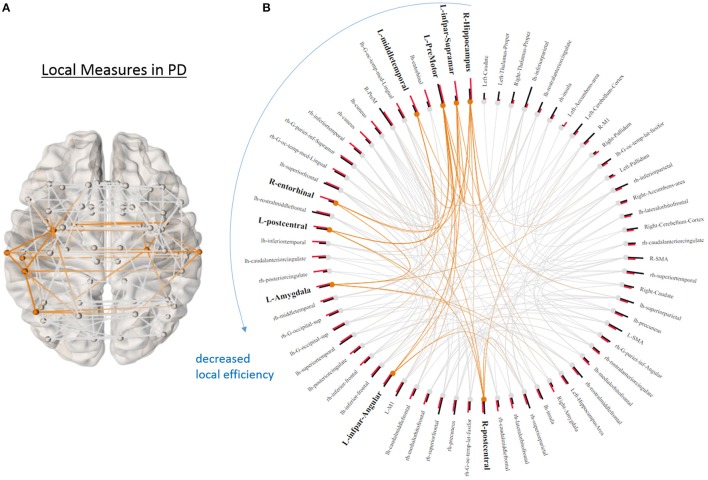
Local graphical measures in PD. **(A)** Partial correlation connectivity patterns with the 6.32th percentiles (strongest 144 connections) are shown in PD. The orange connections indicate the connectivity within the ROIs that present higher local efficiency in PD. **(B)** Local efficiency (red bars) and betweenness centrality (black bars) of each node ranked by local efficiency are shown in a ring diagram. Bold font size indicates the regions that show higher local efficiency in PD (*p* < 0.05, uncorrected). The figure is derived from NeuroMArVL (http://immersive.erc.monash.edu.au/neuromarvl/).

**Figure 4:**
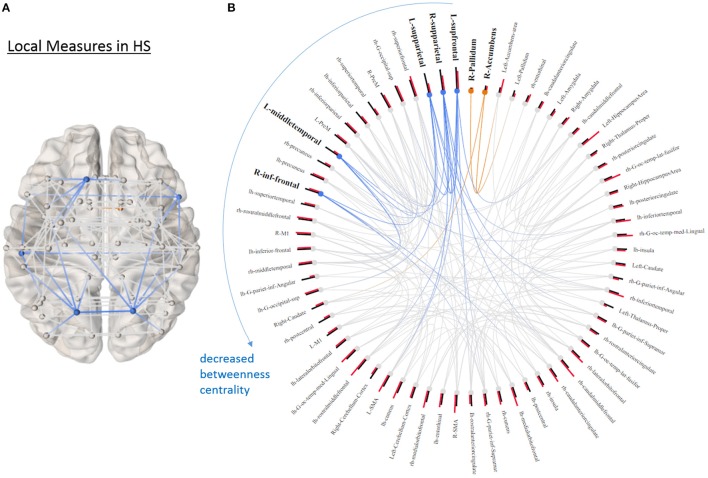
Local graphical measures in HS. **(A)** Partial correlation connectivity patterns with the 6.32th percentiles (strongest 144 connections) are shown in HS. The orange connections indicate the connectivity within the ROIs that distinguish PD and HS with higher betweenness centrality in PD; while the blue ones represent the connections between the ROIs that differentiate two groups with decreased betweenness centrality in PD. **(B)** The ring diagram presents betweenness centrality (black bars) and local efficiency (red bars) of each node ranked by betweenness centrality. Bold and bigger font size indicates the regions that are significantly different between two groups (*p* < 0.05, uncorrected). Except the right pallidum and accumbens, all the regions with bold font show lower betweenness centrality in PD. The figure is derived from NeuroMArVL (http://immersive.erc.monash.edu.au/neuromarvl/).

None of the global graph theoretical measures were different between PD and HS, however, several ROIs showed significantly altered local efficiency and betweenness centrality. Figure 3A shows the average connectivity patterns in PD on a brain template. Color-coded connections indicate the connectivity within the ROIs that distinguish PD and HS. Figure 3B presents the local measures in PD on a ring diagram. The right hippocampus, left supramarginal gyrus, left pre-motor cortex, left middle temporal gyrus, right entorhinal cortex, left postcentral gyrus, left amygdala, left angular gyrus, and right postcentral gyrus all demonstrated higher local efficiency in PD (*p* < 0.05, uncorrected) (Figure 3, Supplementary Figure 2), indicating that functional connectivity was more segregated in PD. Figure 4A highlights the connections that differentiate PD and HS. Figure 4B demonstrates local measures in HS ranked by betweenness centrality in a ring diagram and color-coded the ROIs that were significantly different between two groups. These ROIs showed lower betweenness centrality in PD such as the left superior frontal gyrus, bilateral superior parietal cortices, left middle temporal gyrus, and right inferior frontal gyrus; while the right accumbens area and right pallidum exhibited higher betweenness centrality in PD (Figure 4, Supplementary Figure 3). The changes of betweenness centrality in PD indicated an altered hub organization, where important nodes lost significance and the nodes with a less central role have become more important.

**Figure 5:**
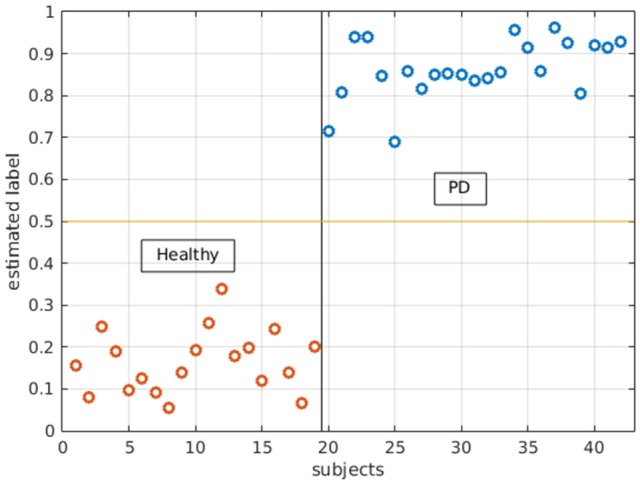
Logistic LASSO distinguishes PD and healthy subjects with all the local measures. In the analysis, PD subjects were assigned 1 and healthy subjects were assigned 0 as their labels. Logistic LASSO predicts the labels based on all local measures (both local efficiency and betweenness centrality). The estimated/predicted labels are accurately categorized into two groups.

With logistic LASSO, the ROIs which showed significant uncorrected p values in the *t*-tests were all important contributors to the separation of PD and HS (Figure 5) except betweenness centrality in the right superior parietal gyrus, which was almost insignificant in *t*-tests (*p* = 0.0415). Moreover, logistic LASSO selected slightly more ROIs that distinguished PD and HS than *t*-tests (Supplementary Table 2).

### Brain-behavior associations

#### Univariate results

**Figure 6:**
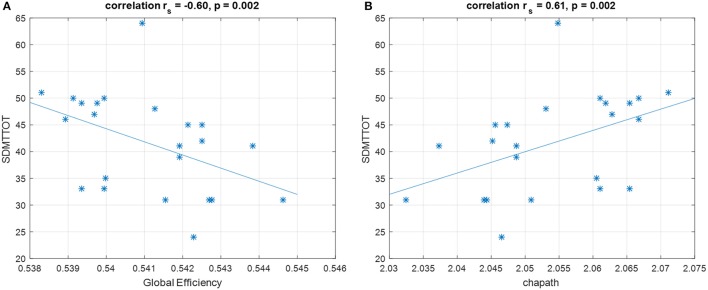
Spearman's correlation shows that SDMT is negatively correlated with **(A)** global efficiency and positively correlated with **(B)** characteristic path length. These correlations survive for Bonferroni correction. SDMTTOT, Symbol Digit Modalities Test total scores; chapath, characteristic path length. ^*^Represents each data point.

In the Spearman's rank correlation analysis, only raw Symbol Digit Modality Test (SDMT) scores were significantly correlated with global efficiency and characteristic path length (*p* = 0.002 and *r*_*s*_ = −0.6, *p* = 0.002 and *r*_*s*_ = 0.61, respectively, Bonferroni corrected for multiple comparisons) (Figure 6). Higher global efficiency was related to lower SDMT scores; while higher characteristic path length (which is the inverse measure of global efficiency) was associated with better performance. There was no significant correlation between all global graphical measures and CP_dem_.

Local graphical measures were not significantly correlated with either the individual cognitive scores or CP_dem_ after correction for multiple comparison.

#### Multivariate results

**Figure 7:**
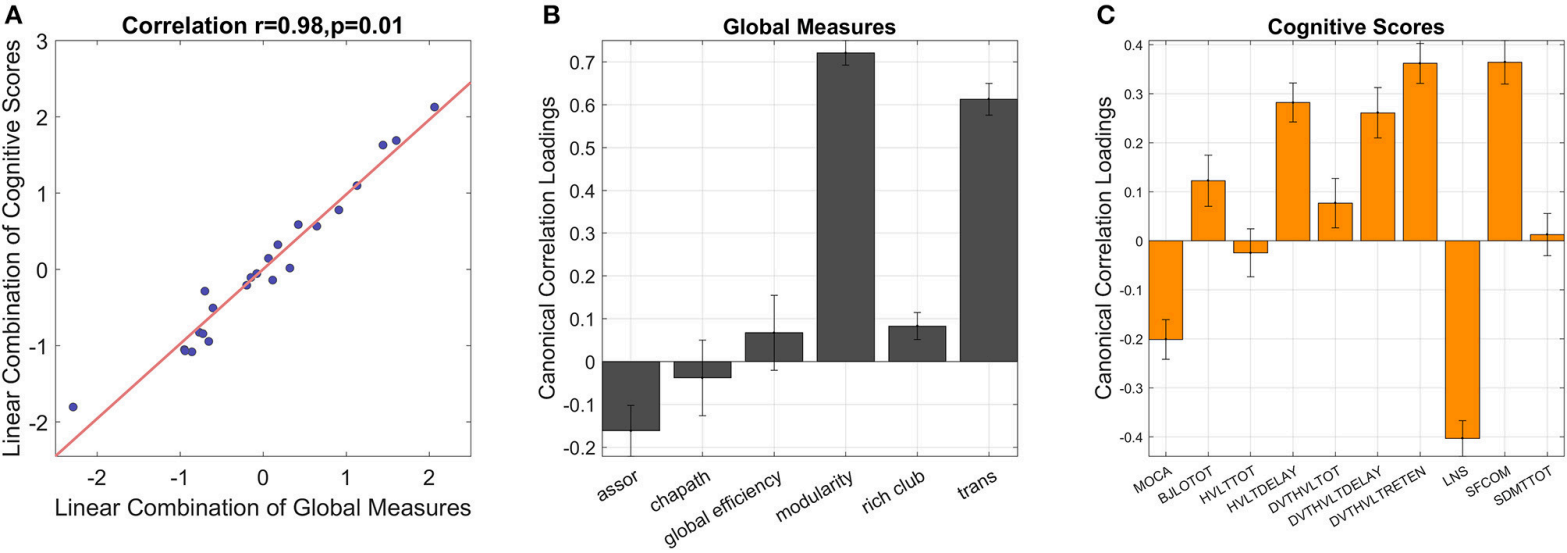
CCA reveals inter-correlations between global graphical measures and cognitive scores [**(A)**, correlation = 0.98, *p* = 0.01]. The error bars indicate 95% confidence interval in leave-one-out cross validation. If the error bars do not cross zero, the variables are recognized as significant. Among global measures **(B)**, assortativity, modularity and transitivity show significant loadings. In the combination of cognitive scores **(C)**, MoCA, BJLO, HVLY delay recall, standardized HVLT total, standardized HVLT retention, LNS, and SF scores contribute significant loadings. assor, assortativity; chapath, characteristic path length; rich club, rich club coefficient; trans, transitivity; MOCA, Montreal Cognitive Assessment; BJLOTOT, Bento Line Orientation Total Score; HVLTTOT, Hopkins Verbal Learning Test-Revised Total Score; HVLTDELAY, HVLT Delayed Recall Score; DVT-HVLTTOTAL, standardized HVLT Total Score; DVT-HVLTDELAY, standardized HVLT Delayed Recall Score; DVT-HVLTRETENTION, standardized HVLT Recognition Trial Score; LNS, raw Letter-Number Sequencing Test Score; SFCOM, Sematic Fluency Test—combination; SDMTTOT, Symbol Digit Modalities Test total scores.

The global graphical measures showed a significant correlation with behavioral scores in CCA (*r* = 0.98, *p* = 0.01, *p* = 0.017 in the permutation test, Figure 7). The variables were considered significant if the error bars did not cross zero. In global measures, assortativity, modularity, rich club coefficient, and transitivity demonstrated significant canonical loadings; while MoCA, BJLO, HVLY delay recall, standardized HVLT total, standardized HVLT retention, LNS, and SF scores appeared influential in the model. Modularity, rich club coefficient, and transitivity were positively correlated with scores of BJLO, HVLY delay recall, standardized HVLT total, standardized HVLT retention, and SF. On the other hand, assortativity was correlated with MoCA and LSN performance.

A CCA model attempting to relate local graphical measures and cognitive scores was not significant.

The correlation between CP_dem_ and CP_gt_ was not significant (*r* = −0.06, *p* = 0.77).

## Discussion

### Cognitive profiles associated with demographics and gender differences

Several studies have utilized CCA to study inter-correlations between behavioral data, cognition, imaging findings, and daily activity in both healthy subjects and disease populations (18, 23–27), indicating that CCA is a useful tool to study relations between two sets of data. In PD, a CCA approach found that working memory, attention, planning, and problem solving were inter-correlated with visuospatial memory and episodic memory in early stage PD and executive function (index of working memory, attention, planning) and visuospatial memory contributed the most to cognitive deficits (26). Although these studies have shown that there are inter-correlations in cognition and disease, gender differences have not been previously observed in PD with CCA approaches.

We found that education supported better cognitive performance in visuospatial, verbal learning, verbal memory, working memory, executive domains as well as information retrieval abilities. Our results are consistent with the concept of *cognitive reserve*, whereby education may induce neuroprotection effects in the setting of neurodegenerative disease (28, 29). Education possibly enhances plasticity and flexibility in a variety of neural circuits, which provides more neural resources supporting brain function (30, 31). However, longitudinal studies have suggested that aging may be a stronger factor regarding cognitive changes than education (29, 32). This is similar to what we found where both education and age showed strong loadings in the CCA model—a pattern that has been reported in healthy subjects as well (27).

Unsurprisingly, we found that depression had a negative effect on cognitive function, consistent with previous research (33); however, we did not have means, due to study design, to probe whether cognitive decline induced depression or depression manifested cognitive dysfunction in PD—an area of ongoing debate (34).

The gender differences that we discovered have been observed previously in PD, but the differences in cognition have not been extensively studied and results of non-motor symptoms have been inconsistent (35, 36). In general, female subjects tend to perform better on many cognitive tests in older healthy subjects (24). However, a study with 1,700 individuals with PD reported that motor-symptoms were not different between male and female, but female subjects fared better in non-motor aspects which included SDMT performance and daily functioning (36). Moreover, male subjects have faster decline of cognitive abilities in verbal, letter, and category fluency tests; while females tend to have worse visuospatial abilities (37, 38). Our results also demonstrated that females performed better in SDMT, verbal fluency tests, and overall cognition measured by MoCA, but presented worse visuospatial function, consistent with previous research. The purported mechanisms of gender differences in cognition are unclear, but it has been postulated that estrogen may impart neuroprotective effects on dopaminergic pathways (35). Our previous work similarly concluded that female subjects with multiple sclerosis also showed relatively preserved cognition in several domains (18), partially supporting that estrogen serves overall neuroprotective effects robust to the exact nature of pathology.

Surprisingly, the commonly used measure of overall disease severity, UPDRS, did not show significant loadings in the model relating cognitive performance to clinical measures, which implied that motor assessment did not directly reflect cognitive decline beyond that predicted by other variables. However, when UPDRS was replaced by the individual sub-scores, axial and tremor symptoms did show significant loadings, contributing to the anti-correlated patterns between aging/disease severity/comorbidity and cognitive performance (Supplementary Figures 4, 5), demonstrating that axial and tremor symptoms were related to worse cognitive performance in PD (4, 39). The results may be due to partial overlap between motor and cognitive pathways and systems (40).

### Changes of brain organization in PD

Overall brain organization can be thought of as a balance between integration and segregation, in which the former facilitates information integration across the whole brain; while the latter enables efficient information transfer within individual networks (8). This organization allows the brain to function in an economical way by reducing the wiring cost of linking anatomically segregated regions (41). In addition, hub regions—the cortical areas that play a central role in the networks—support information integration in many cognitive functions as well as neuronal coupling between networks (15). These principles together maintain brain function and support cognition in healthy subjects; in the diseased brain, this delicate balance is altered and fails to sustain normal functioning (14, 42, 43).

Some studies have reported altered functional organization implied by graph theoretical measures of rsFC in PD. For example, PD subjects with MCI have increased measures of segregation such as modularity and clustering coefficient, and these measures in frontal areas are both negatively and positively correlated with cognition, suggesting that functional connectivity in the frontal regions has been adapted to support cognitive functions (13). Although other studies reported decreased clustering coefficient and local efficiency (44), implying FC might be less segregated, differences in methodology should be taken into account. Increased local efficiency has been observed in PD in another study, further supporting the idea that information transfer is stronger within local sub-networks as opposed to the global network system in PD (45).

Our results of local graph theoretical measures indicate increased segregation of resting-state functional connectivity (rsFC) in PD. ROIs across temporal, occipital, parietal, motor association, and sensory regions had higher local efficiency in PD. Interestingly, most of the ROIs that had higher local efficiency also showed significant differences in measures indicating network segregation in PD (Figure 3). We also observed hub changes in our data, with many ROIs demonstrating decreased betweenness centrality (i.e., an index of hub and measures how important a given node is) such as the superior frontal gyrus, superior parietal gyrus, middle temporal gyrus, and inferior frontal gyrus, which have been previously suggested as functional hubs (15) (Figure 4). Although the results were not significant after controlling for multiple comparisons when assessed in univariate fashion, there was still a trend that these hub regions were affected compared to non-hub regions. In addition, the logistic LASSO model demonstrated that these altered local measures were clearly able to distinguish the PD from the HS group. Thus our findings are consistent with the *vulnerability of hubs* in neurological disease populations (14, 43). Only two ROIs (the right accumbens and pallidum) in this PD population demonstrated increased betweenness centrality and these ROIs in HS showed the smallest values (Figure 4).

The increase in connectivity in the right accumbens and pallidum that we observed is intriguing. The nucleus accumbens (NAc) receives dopaminergic projections from the ventral tegmental area (VTA) and substantia nigra (SN) regions and projects to several deep gray matter areas which include globus pallidus; therefore, the NAc has been hypothesized to be associated with the nigrostriatal and mesolimbic systems (46) and linked to reward behavior (47). In PD, the NAc receives less dopamine projection from the VTA and SN and the globus pallidus receives output from the NAc, so the increased FC is perhaps surprising. The PD patients in this study were relatively mildly affected, and likely capable of significant amount of compensation. Thus NAc and pallidum may up-regulate their overall connectivity to maintain function.

Unlike previous studies which reported changes in global measures such as modularity (13), we did not observe differences in global indices even before correction for multiple comparisons but found changes in local measures. Again, as our cohort was relatively mildly affected, the limited damage in local regions might not be severe enough to impact global measures. Local FC might be more sensitive to pathological and physiological features and potentially serve as biomarkers than global measures at early disease stages.

The logistic LASSO model differentiating HC from PD from graph theoretical measures selected more ROIs than univariate *t*-tests. This implies that while individual ROIs may only be mildly discriminative, collectively they provide a robust way to distinguish between groups. Although not all ROIs selected by the logistic LASSO model were hubs, the results indicated an altered network phenomenon may be initiated at hub regions.

### Reduced functional integration and increased functional segregation correlated with better cognitive performance in PD

The relations between FC, graph theoretical measures, and cognition have gained attention lately in network neuroscience and clinical studies. Several graphical measures have been associated with intelligence, working memory, and executive functions in healthy subjects (10, 11, 48), yet research in PD has been limited to a few studies. With a correlation approach, a study reported that hub organization in PD was more related to dopaminergic medication dosage rather than cognitive functions (17). Although the study did not rule out the associations between hubs and cognitive functions that a simple correlation method might not be able to catch, sophisticated statistical analyses are required to explore complex relations between cognition and FC. With a linear regression analysis, Baggio et al. discovered that increased local measures in PD with MCI, such as clustering coefficient, local efficiency, and modularity in the frontal areas, were correlated with worse attention and executive functions (13). Overall, in PD, more evidence of the associations between cognitive functions and FC is needed to underline the neural mechanism of cognition and how the disease affects behavior.

In this study, we utilized both univariate (correlation) and multi-variate (CCA) approaches to explore the relations between graphical measures and cognition in PD. With a univariate approach, we observed strong correlations between the measures of integration (i.e., global efficiency and characteristic path length) and SDMT scores in PD. The performance of SDMT requires attention, processing speed, scanning abilities, and engages several brain regions including the occipital cortex, middle frontal gyrus, precuneus, superior parietal lobes, and cerebellum (49) and has been proven as a robust tool to detect cognitive impairments in healthy aging and neurological disorders (50–52). Therefore, it would be reasonable to assume that higher functional integration is related to better SDMT performance. However, our results demonstrated the opposite trend, whereby higher functional integration (i.e., higher global efficiency and lower characteristic path length, as they are inversely related) was related to poor performance of SDMT. Such paradoxical relations may be potentially due to disease effects, leading toward a more segregation-oriented brain organization in order to respond to cognitive demand. This is partially consistent with prior reports, demonstrating more segregated FC in frontal areas related to attention/executive function in PD (13). We suggest that “pathological resonance” in the basal ganglia–cortical network, previously described in PD (53), may result in excessive *regional* integration but *global* segregation.

Similarly, we found that segregation-oriented brain organization was related to better cognitive performance with a multivariate approach. Modularity and transitivity, which are both measures of segregation, were significantly correlated with better performances in line orientation, verbal learning, and sematic fluency tests. This pattern indicated that better visuospatial, verbal learning and memory functions, and executive skills were correlated with segregated brain organization in PD. In addition, rich club coefficient, an indication of rich club structure which is thought to facilitate cognitive processes (54), was also related to better performance of the above-mentioned tests, further emphasizing the important role of rich club structure in cognition. On the other hand, assortativity, which shows the tendency of nodes with similar connectivity to link with each other, was correlated with overall cognitive function as well as memory, attention, and mental manipulation skills. However, as assortativity was ultimately a correlation coefficient between node degrees (definition is shown in Supplementary Table 1) and the measure was small in all subjects (maximum value = 0.09, minimum value = −0.1), we did not think this measure itself represented very meaningful information in this cohort even though it was correlated with MoCA and LNS scores. Therefore, taken together, we interpreted that multivariate approach also revealed similar findings as univariate analysis but with greater robustness, whereby better cognitive function was related to segregated brain networks as well as rich club characteristics in PD.

In this study, we did not report significant relations between local measures and cognitive performance. This is somewhat surprising since the local graphical measures could be used to distinguish between subject groups, while the global measures could not. The reason could be that the overall, global brain organization is more related to cognitive performance, while smaller, more subtle changes in local network characteristics may be related to other non-cognitive differences between groups (e.g., related to motor function).

The optimal balance between brain segregation and integration for higher-order cognitive function remains a source of debate, with previous research arguing for more of one or the other (10, 48). Between-network communication (i.e., integration) may be important for working memory (10), but executive functions may require more nodal FC (i.e., segregation) (48). The delicate balance of segregation/integration may relate to the balance between focusing on internal brain states vs. external sensory input (55). Sensory input from several cortices can send signals to the prefrontal cortex, which integrates this information with internal brain states, with the resultant output transferred to subcortical and motor association areas to executive the action. Presumably, during processing of input and output in the prefrontal regions, integration is required in order to communicate with different brain regions; while processing internal states, redundant and unnecessary regions are excluded in order to focus on the processes within the prefrontal cortex. We propose that the disease may have preferentially affected integration between remote regions rather than internal processing, consistent with the “pathological resonance” concept alluded to above. This suggests that in PD the prefrontal cortex especially will be excessively robust to sensory driven external inputs, resulting in impaired set shifting seen in PD in patients with frontal lobe damage (56).

### Unique cognitive patterns and graphical measures represented different aspects in PD

In this study, we used CCA to explore relations between (1) demographics/cognition and (2) graphical measures/cognition in PD. As a result, two unique cognitive profiles were revealed and each of them provided different but valued information. The cognitive profile associated with demographics showed that female sex and higher education were related to better cognitive performance. The other cognitive profile associated with graphical measures illustrated the correlations between segregated brain organization and better cognitive function. These two cognitive profiles were not the same and they were not related to each other either. If they were highly correlated, this would have meant that graphical measures do not provide complementary information to demographics. The fact that two cognitive profiles were distinct further supports the value of utilizing graphical measures to study rsFC and cognition.

We discovered differences between PD and HS with *local measures* rather than global measures. Yet local measures did not show correlations to cognition, rather, *global measures* exhibited inter-correlated patterns to cognitive performance. Therefore, we propose that local measures might be more sensitive to subtle functional disruptions as biomarkers; while global measures are more related to cognitive performance as cognition is a network phenomenon (57).

### Limitations

There are several limitations to our study. First, due to motion and image artifacts, eight subjects had to be removed from the connectivity analysis and the sample size was relatively small. For advanced statistical approaches, including machine learning methods, a bigger sample size will likely be needed. Moreover, as we focused on the baseline data in PPMI database, the subjects tended to be only mildly affected and were in the early stages of the disease. This could be the reason why we did not observe significant global differences between PD and HS and the altered local brain organization was limited with strict statistical criteria. In addition, most of the control subjects in PPMI database went through fMRI and T1-weighted image scans at different visits (often 2–3 years apart). In order to avoid potential morphometry changes in an elder population, we included healthy subjects in our center. Future studies should include the data acquired in the same scanner with same parameters in order to reduce variability. Finally, there is a growing trend to investigate the relations between cognition and *dynamic* functional connectivity measures. Due to sample size, we did not perform such analysis. Therefore, there was no evidence to evaluate whether (1) PD presented altered dynamic functional connectivity or (2) PD pathologies impacted the relations between dynamic information coordination and cognitive functions.

## Conclusions

In this study, we explored the relations between cognitive performance and demographics/clinical data in PD, demonstrating that female sex and education supported cognitive performance, and age and depression were associated with poorer performance. With a graph theoretical approach, we demonstrated that rsFC was more segregated in PD across regions. Additionally, PD subjects demonstrated hub vulnerability. Increased connectivity in the nucleus accumbens and pallidum suggested possible compensation for PD pathologies in mildly affected individuals. Individually, hubs demonstrated weak alterations, but collectively more regions (both hubs and non-hubs) exhibited stronger power to discriminate PD and HS. Finally, with both univariate and multivariate analyses, we concluded that visuospatial, verbal learning and memory, attention, processing speed, and executive abilities were associated with segregated FC and rich club structure in PD, possibly related to pathological synchrony in basal ganglia structures as well as the links between cognitive function and rich club organization in a diseased cohort.

## Ethics statement

The PD data included in this study are from the Parkinson's Progression Markers Initiative (PPMI) database. For the ethics statements, please see the detailed in http://www.ppmi-info.org. The ethics approval for HS was issued by the University of British Columbia's Research Ethics Board.

## Author contributions

S-JL wrote the manuscript and performed all analyses. TB and SG contributed to imaging preprocessing and further analyses as well as constructing the ideas for the manuscript. MM supervised the study and contributed to the writing.

### Conflict of interest statement

The authors declare that the research was conducted in the absence of any commercial or financial relationships that could be construed as a potential conflict of interest.
