# Supplementary material for: Cognitive Profiles and Hub Vulnerability in Parkinson's Disease
Source: Front Neurol. 2018 Jun 20;9:482. doi: 10.3389/fneur.2018.00482 (PMC6019441; doi:10.3389/fneur.2018.00482)
Supplement: Supplementary file 1 [file Presentation_1.PDF]

Supplementary Material

Cognitive Profiles and Hub Vulnerability in Parkinson's Disease

Sue-Jin Lin, Tobias R. Baumeister, Saurabh Garg, Martin J. McKeown*

*** Correspondence:** Martin J. McKeown, email: martin.mckeown@ubc.ca

1 Supplementary Figures and Tables

1.1 Supplementary Tables

	measures	definition
global measures	global efficiency	the average inverse shortest path length in the network (measure of integration)
	transitivity	the ratio of triangles to triplets in the network (measure of segregation)
	modularity	it quantifies the degree to which network may be subdivided into clearly different groups (measure of segregation)
	assortativity	correlation coefficient between the degrees of all nodes on two opposite ends of a link (nodes link to other similar nodes, measure of resilience)

	characteristic path length	the average shortest path length in the network (measure of integration)
	rich club coefficient	the fraction of edges that connect nodes of degree *k* or higher out of the maximum number of edges that such nodes might share (in this study k = 6)
local measures	betweenness centrality	the fraction of all shortest paths in the network that contain a given node (measure of centrality/hub)
	local efficiency	the global efficiency computed on the neighborhood of the node (measure of segregation)

Table 1. The definition of graph theory measures computed in the study.
(references: https://sites.google.com/site/bctnet/ and Rubinov & Sporns 2010[1])

1. Rubinov M, Sporns O. Complex network measures of brain connectivity: Uses and interpretations. Neuroimage. 2010;52:1059–1069.

t-tests		logistic Lasso	
local efficiency	betweenness centrality	local efficiency	betweenness centrality
5, left amygdala	8, left superior frontal gyrus	2, left pallidum	**8, left superior frontal gyrus**
18, left middle temporal gyrus	18, left middle temporal gyrus	**5, left amygdala**	9, left rostral middle frontal gyrus
24, left postcentral gyrus	28, left superior parietal gyrus	6, left insula	12, left lateral orbitofrontal gyrus
29, left angular gyrus	36, right pallidum	7, left accumbens	16, left entorhinal gyrus
30, left supramarginal gyrus	41, right accumbens	14, caudal anterior cingulate cortex	**18, left middle temporal gyrus**
34, left premotor area	45, right inferior frontal gyrus	**18, left middle temporal gyrus**	**28, left superior parietal gyrus**
38, right hippocampus area	*62, right superior parietal gyrus*	19, left superior temporal gyrus	30, left supramarginal gyrus
50, right entorihinal gyrus		**24, left postcentral gyrus**	35, right thalamus
58, right postcentral gyrus		**29, left angular gyrus**	**36, right pallidum**
		**30, left supramarginal gyrus**	38, right hippocampus
		33, left SMA	**41, right accumbens**

	**34, left premotor area**	**45, right inferior frontal gyrus**
	**38, right hippocampus area**	54, right superior occipital gyrus
	41, right accumbens	
	**50, right entorihinal gyrus**	
	**58, right postcentral gyrus**	
	60, right precuneus	
	64, right supramarginal gyrus	

Table 2. Logistic LASSO and t-test results. All the significant ROIs from t-tests are shown in logistic LASSO (in bold) except the right superior parietal gyrus (in italic). Logistic LASSO revels more ROIs that are important to distinguish PD and HS. The number before each ROI represents the index of the region in the analysis.

1.2 Supplementary Figures

Supplementary Figure 1.

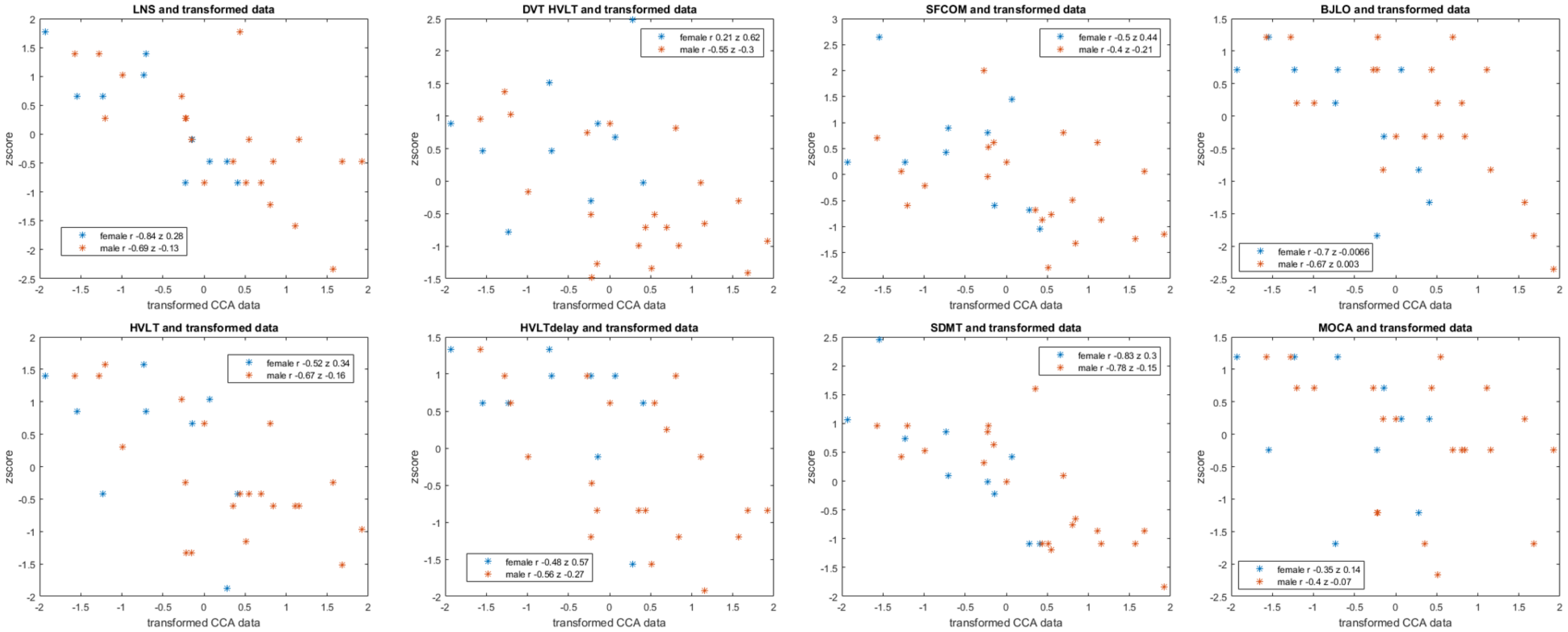

The figure illustrates canonical loadings (correlation between transformed CCA data and raw scores of CCA input) of the cognitive variables which show significant impacts in the CCA model. In each score, blue dots represent female data and red dots are male data. Average canonical loadings (indicated as **r** in the legend) are calculated as well as average cognitive z-scores in both groups. Except BJLO (the upper right corner), female subjects show higher average cognitive scores, indicating better performances.

Supplementary Figure 2.

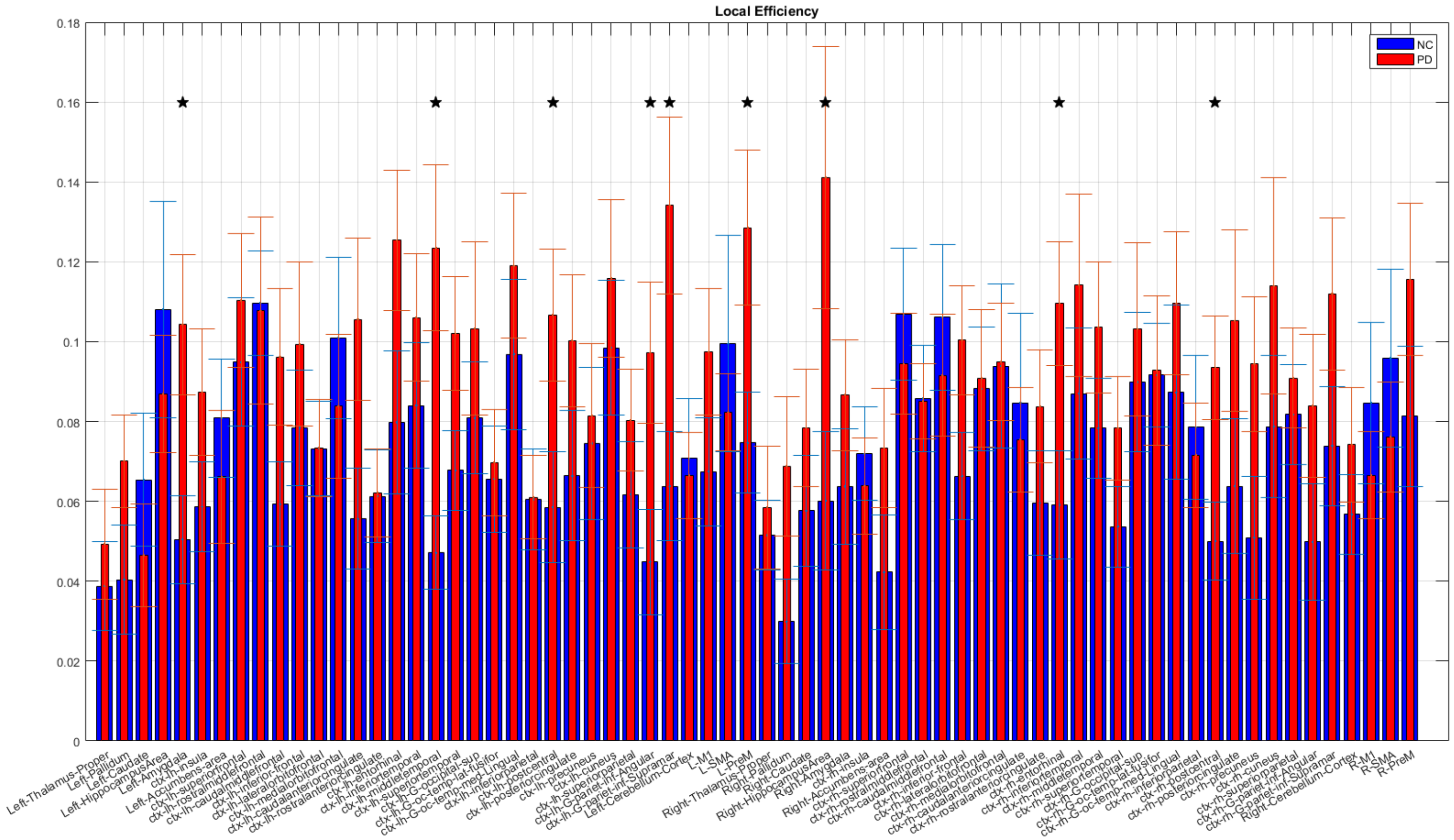

Nine ROIs show higher local efficiency in PD indicated with black stars ($p<0.05$, uncorrected). These ROIs are the left amygdala, left middle temporal gyrus, left postcentral gyrus, left angular gyrus, left supramarginal gyrus, left pre-motor area, right hippocampus, right entorhinal cortex, and right postcentral gyrus.

Supplementary Figure 3.

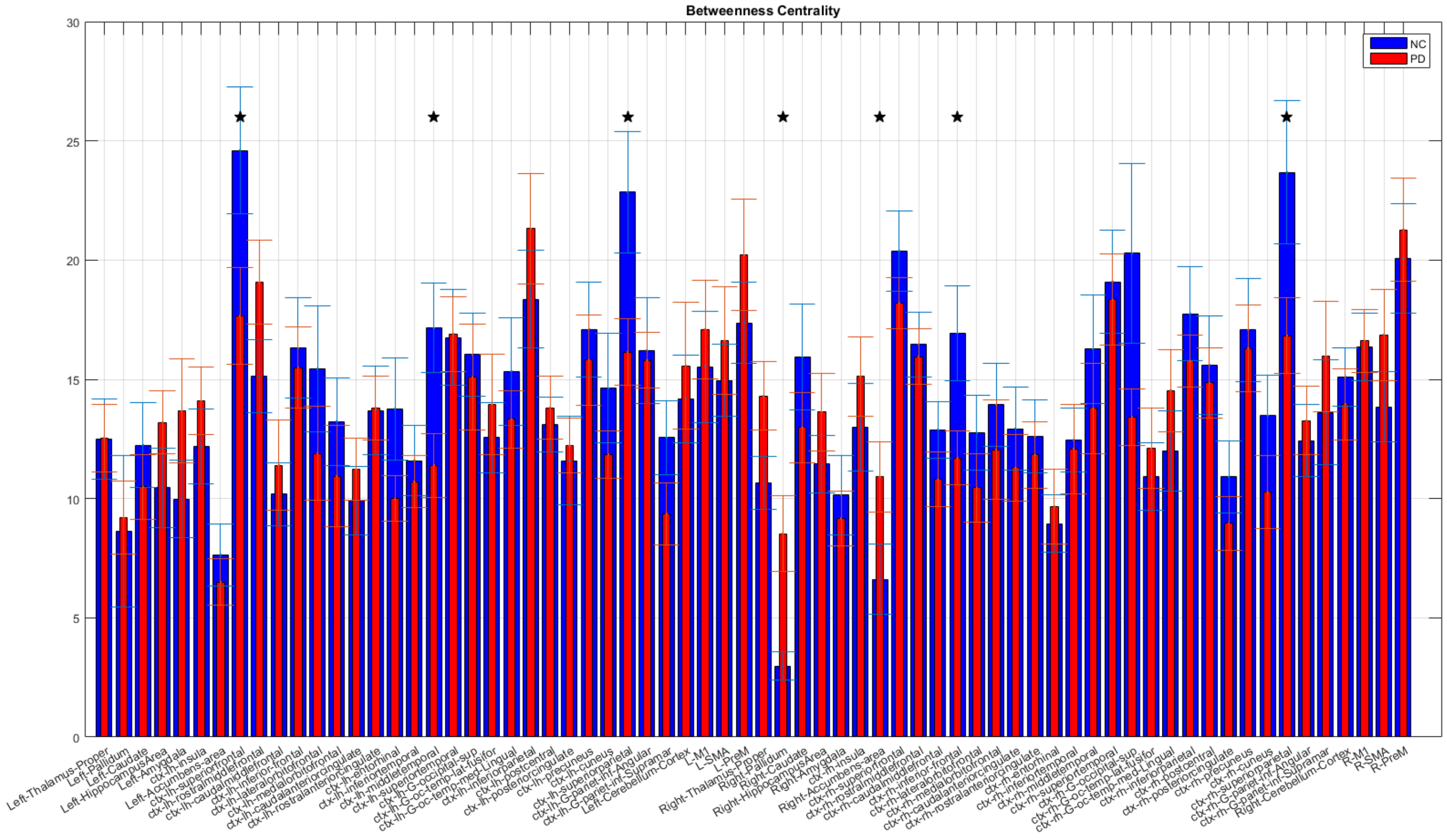

Seven ROIs show altered betweenness centrality in PD, which are indicated with black starts ($p<0.05$, uncorrected). The right pallidum and right accumbens areas show increased betweenness centrality; while the rest ROIs show decreased values such as the left superior frontal gyrus, left middle temporal gyrus, left superior parietal gyrus, right inferior frontal gyrus, and right superior parietal gyrus.

Supplementary Figure 4.

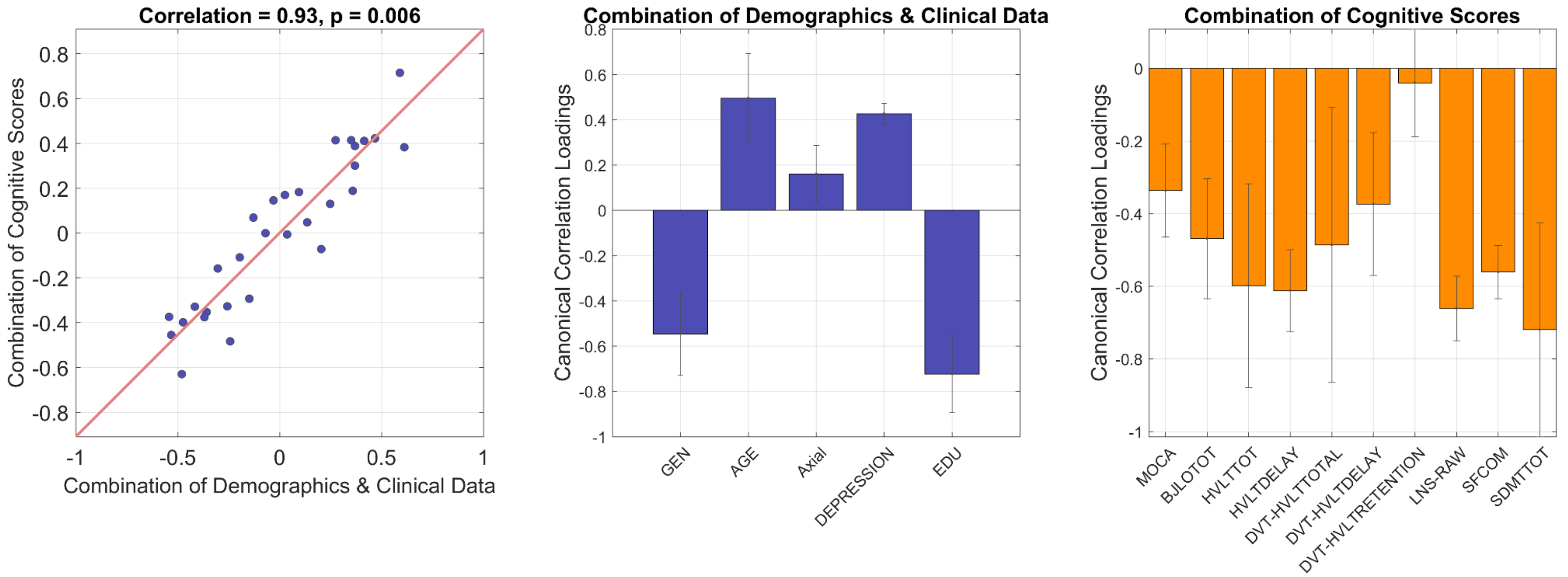

UPDRS full score does not show significant loadings in the previous model, but replacing it with axial symptom score allows disease severity to demonstrate significant influence, whereby higher age, more severe axial and depression symptoms are anti-correlated with better cognitive performance (correlation coefficient=0.93 and p=0.006).

Supplementary Figure 5.

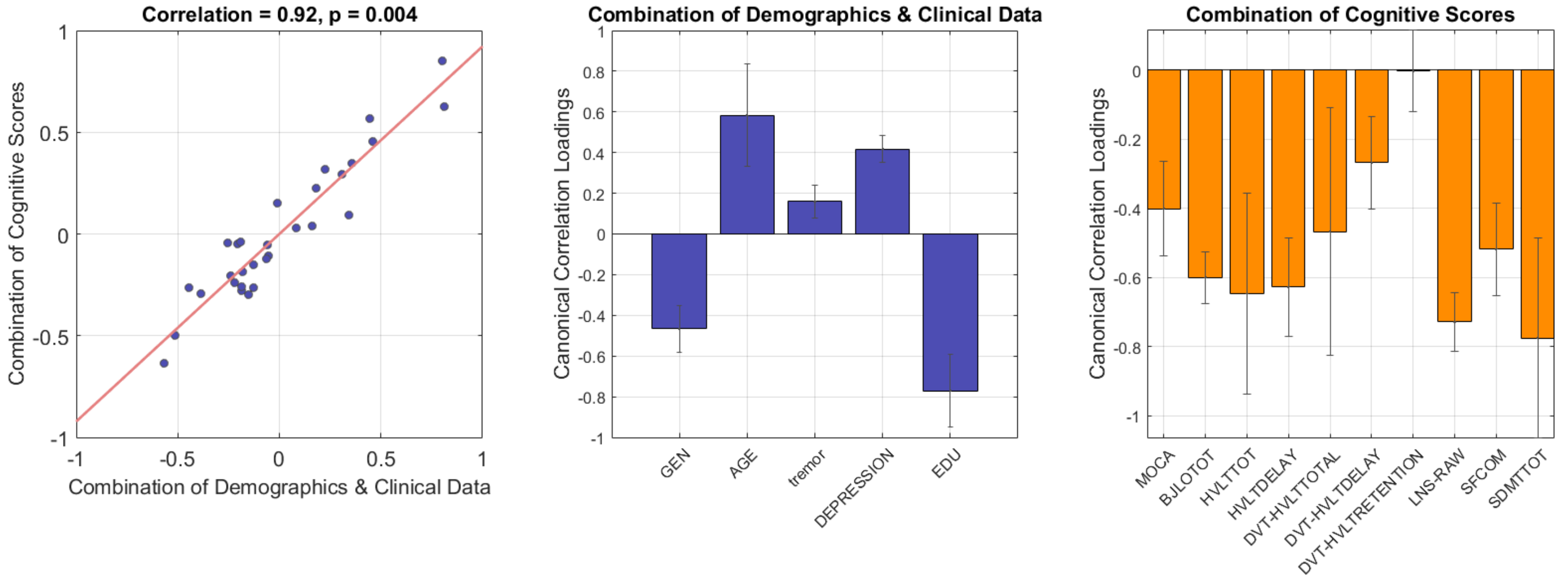

Replacing UPDRS full score with the sub-score of tremor demonstrates a significant mode, whereby higher age and more severe tremor and depression symptoms are anti-correlated with better cognitive performance (correlation coefficient=0.92 and p=0.004).
